# Heritage and Health – Improving the Wellbeing of the Older People in the New Forest Through Understanding the Barriers to Access and the Creation of a Heritage and Nature Group

**DOI:** 10.1192/bjo.2025.10463

**Published:** 2025-06-20

**Authors:** Jennifer Wyllie, Laura Pridmore

**Affiliations:** 1 New Forest National Park Authority, Lymington, United Kingdom; 2 Hampshire County Council, Winchester, United Kingdom; 3 Hampshire and Isle of Wight Healthcare NHS Foundation Trust, Tatchbury, United Kingdom

## Abstract

**Aims:** Hampshire has an older population structure, compared with the national average, with increases predicted amongst the older population, aged 75 years and older. This group is more susceptible to social isolation, particularly those living in towns of the New Forest. We therefore are focusing on a project aimed at the older population, particularly men, in the town of Lyndhurst.

Our aims are threefold: to understand what current nature-based activities are on offer for older people in the New Forest, to appreciate the barriers for engagement to these and lastly through insights with this group to set up a heritage and nature group to try to tackle isolation and improve physical activity levels.

**Methods:** We have held several insight groups with local community groups to better understand their route to engage in nature-based activities and what barriers they faced. Additionally, we have met with social prescribers in the New Forest PCN to better understand isolation in the elderly population.

We have arranged two focus groups at the Heritage centre in Lyndhurst to better understand the current community offering and to also help us launch our pilot heritage and nature group running at the centre.

**Results:** Common themes were highlighted as barriers to engagement in nature activities including poor public transport, reduced mobility, caring responsibilities, lack of diversity of activities as well as the intangible barrier of ‘it’s not for me’. Digital barriers further negatively impact those who are unable to access information about groups in the local area and space can be difficult to find for groups to run such as for the Lyndhurst shed.

**Conclusion:** Our work so far has highlighted that older people are more susceptible to isolation and that it is more of a concern in towns rather than rural areas in the New Forest. We have identified that there is a gap in heritage and nature-based activities on offer in the New Forest, which would encompass several aspects of the 5 ways to wellbeing such as connecting with others, being active (brief history walk) and learning new things. By involving local older people in the set-up of the group through focus groups we have been better able to address any barriers they may have to engage in nature and heritage activities. This project adds to the existing evidence of the positive interaction between heritage and wellbeing.

